# LncRNA136131 suppresses apoptosis of renal tubular epithelial cells in acute kidney injury by targeting the miR-378a-3p/Rab10 axis

**DOI:** 10.18632/aging.204036

**Published:** 2022-04-28

**Authors:** Zhifen Wu, Jian Pan, Jurong Yang, Dongshan Zhang

**Affiliations:** 1 Department of Nephrology, The Third Affiliated Hospital of Chongqing Medical University, Chongqing, People’s Republic of China; 2 Department of Emergency Medicine, Second Xiangya Hospital, Central South University, Changsha, Hunan, People’s Republic of China; 3 Emergency Medicine and Difficult Diseases Institute, Second Xiangya Hospital, Central South University, Changsha, Hunan, People’s Republic of China

**Keywords:** AKI, lncRNA, miR-378a-3p, Rab10, apoptosis

## Abstract

The pathogenesis of acute kidney injury (AKI) is not fully understood. To date, the exact role and regulatory mechanism of long non-coding RNA (lncRNA)136131 in AKI remains unclear. Here, we demonstrate that lncRNA136131 in BUMPT cells is induced by antimycin A. Furthermore, after incubating BUMPT cells in antimycin for two hours, lncRNA136131 prevented BUMPT cell apoptosis and cleaved caspase-3 expression. Mechanistically, lncRNA136131 sponged miR-378a-3p and then increased the expression of Rab10 to suppress apoptosis. Finally, I/R-induced decline of renal function, tubular damage, renal tubular cells apoptosis, and the upregulation of cleaved caspase-3 were aggravated by lncRNA136131 siRNA. In contrast, this effect was attenuated by the overexpression of lncRNA136131. In conclusion, lncRNA136131 protected against I/R-induced AKI progression by targeting miR-378a-3p/Rab10 and may be utilized as a novel target for AKI therapeutics.

## INTRODUCTION

Acute kidney injury (AKI), a common and critical condition with a high mortality rate and a higher risk of chronic kidney disease (CKD) progressing to end-stage renal disease (ESRD), is mainly caused by ischemia/reperfusion (I/R), sepsis, and various nephrotoxins [1–4]. To date, in addition to dialysis, no effective intervention and treatment for AKI has been established [5, 6]. Hence, it is essential to elucidate the regulatory mechanism of AKI occurrence and development.

Long non-coding RNAs (lncRNAs), which are greater than 200 nucleotides in length [7], have been reported to take part in the progression of AKI [8]. For example, lncRNA 16406, lncRNA LOC105374325, lncRNA PRNCR1, Hotair, and TCONS 00016233 mediate sepsis or cisplatin-induced progression of AKI by promoting renal tubular cells apoptosis [9–13]. Usually, most lncRNAs sponge microRNAs to regulate the expression level of target genes [14, 15]. LncRNA ENSMUST00000136131 (LncRNA136131) is located on chromosome 11 (chr11:83,466,144-83,467,968); however, its biological function, regulatory mechanism, and disease correlation remain unclear. Here, we hypothesize that lncRNA136131 participates in the development of ischemic AKI via miRNA regulation.

In this study, we found that lncRNA136131 in BUMPT cells was induced by incubating with antimycin. LncRNA136131 prevented I/R-induced apoptosis of BUMPT cells. Mechanistically, lncRNA136131 targeted miR-378a-3p and then increased Rab10 expression. *In vivo,* I/R-induced AKI was aggravated by lncRNA136131 siRNA, which was attenuated by lncRNA136131 overexpression. Taken together, these findings suggest that the overexpression of lncRNA136131 prevents I/R-induced AKI progression via the miR-378a-3p/Rab10 axis.

## RESULTS

### The expression of lncRNA136131 is induced by I/R treatment of BUMPT cells

**Figure 1:**
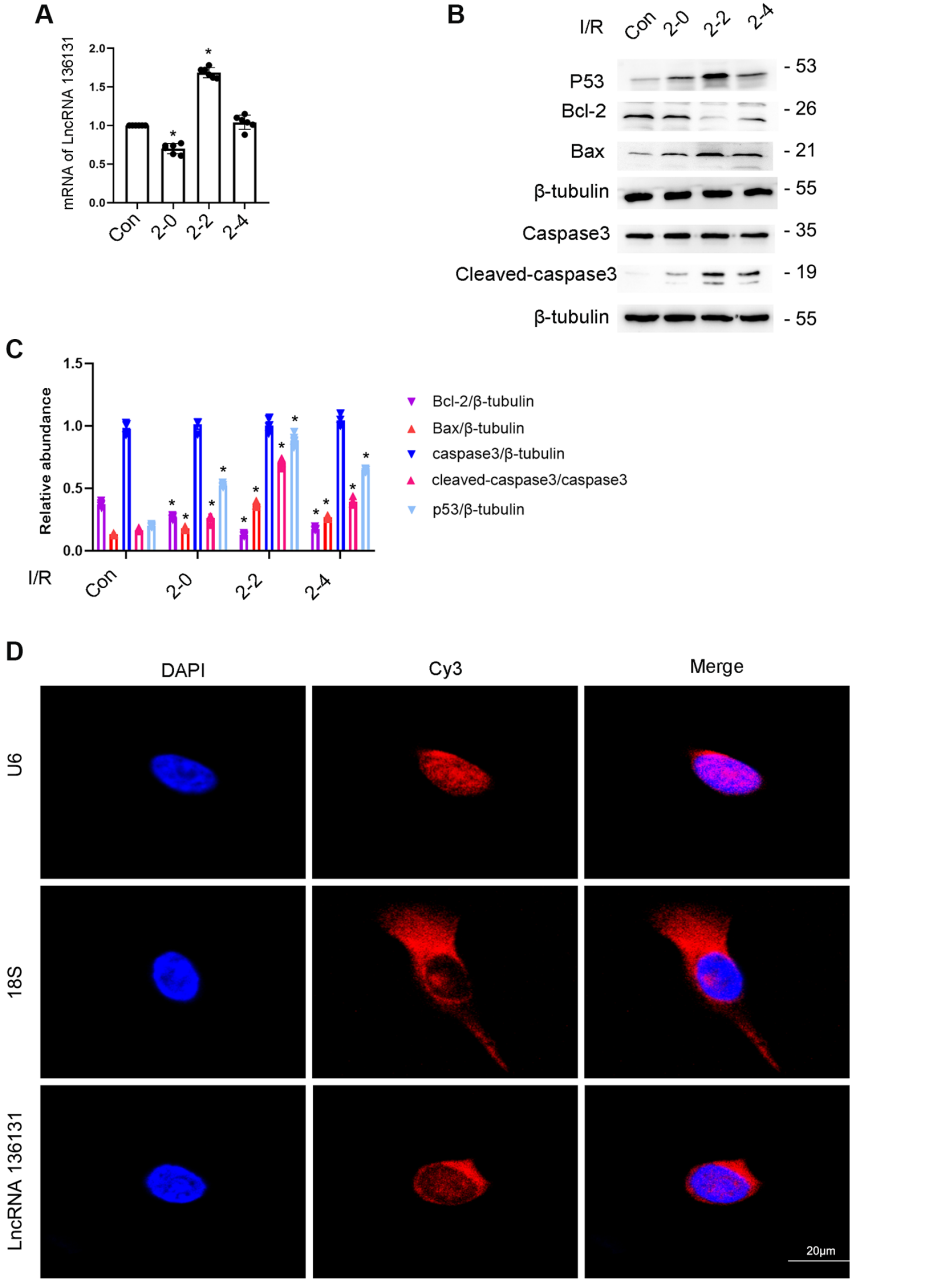
**I/R Induced the expression of lncRNA136131 in BUMPT cells.** BUMPT cells were subjected to ischemic (I, 2 hours)/reperfusion (R, 0, 2, and 4 hours) treatment. (**A**) RT-qPCR analysis of the expression of lncRNA136131. (**B**) The immunoblot analysis of the expression of caspase 3, cleaved-caspase3, Bax, p53 and Blc-2. (**C**). Densitometric measurement of western blot bands of them. (**D**) The RNA-FISH detection of Intracellular localization of lncRNA136131 in BUMPT cells. U6 and 18S were used as control of nucleus and cytoplasm markers, respectively. Data are expressed as mean ± SD (*n* = 6). ^*^p < 0.05, I/R group versus control group.

In this study, BUMPT cells were treated with I (2 h)/R (0–4 h) injury. RT-qPCR analysis demonstrated that the expression of lncRNA136131 induced by ischemic injury gradually increased at 0 hours after reperfusion and peaked 2 h after reperfusion, and finally decreased 4 h after reperfusion (Figure 1A). The expression profile of p53, Bax and cleaved caspase-3 coincided with lncRNA136131 expression, whereas that of Bcl-2 was inversely correlated to lncRNA136131, (Figure 1B, 1C). Furthermore, RNA FISH localized lncRNA136131 to the cytoplasm of BUMPT cells, which coincides with the cytoplasmic marker 18S rRNA but not the nuclear marker U6. These results show that I/R induced the expression of lncRNA136131 (Figure 1D and Supplementary Figure 1).

### LncRNA136131 siRNA enhances I/R-induced BUMPT cell apoptosis

**Figure 2:**
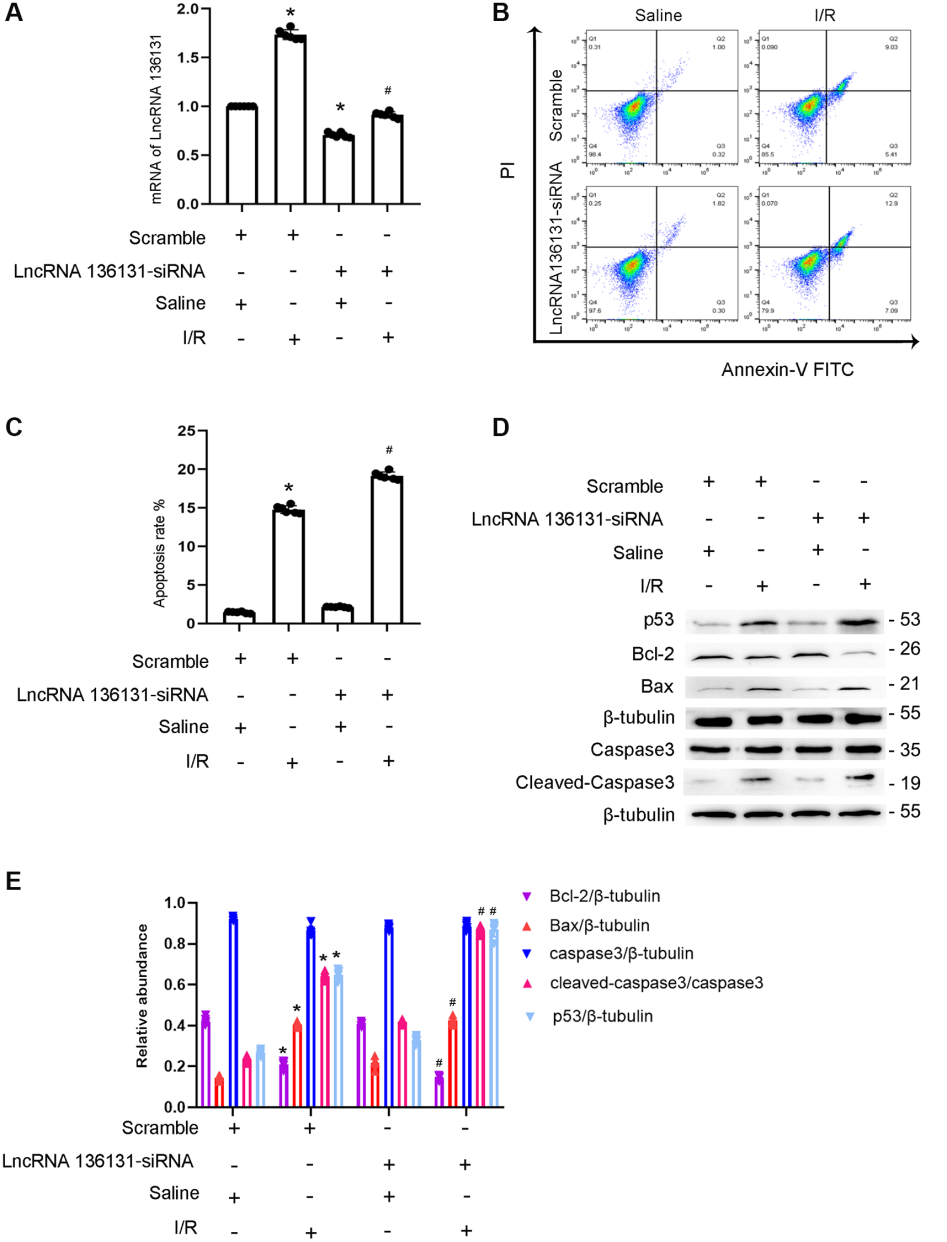
**lncRNA136131 knock down aggravated the I/R-induced BUMPT cells apoptosis and the expression of cleaved-caspase3.** BUMPT cells were transfected with 100 nM lncRNA136131 siRNA or scramble, and then treated with or without I(2 hours)/R(2 hours) injury. (**A**) RT-qPCR analysis of the expression of lncRNA136131. (**B**) FCM analysis of BUMPT cells apoptosis. (**C**) Analysis of apoptosis rate (%). (**D**) The immunoblot analysis of caspase 3, cleaved-caspase3, Bax, p53 and Blc-2. (**E**) The gray analysis between them. Data are expressed as mean ± SD (*n* = 6). ^*^p < 0.05, I/R with scramble or lncRNA136131 siRNA group versus scramble group; ^#^*p* < 0.05, I/R with lncRNA136131 siRNA group versus I/R with scramble group.

We further explored the role of lncRNA136131. The results indicated that lncRNA136131 siRNA effectively silenced its expression under basic or I/R conditions (Figure 2A). Flow cytometry (FCM) analysis showed that lncRNA136131 siRNA notably enhanced I/R-induced apoptosis of siRNA-transfected BUMPT cells (Figure 2B, 2C), which was further verified by immunoblotting of cleaved caspase-3,Bax, p53 and Bcl2 (Figure 2D, 2E). These findings suggest that endogenous lncRNA136131 plays an anti-apoptotic role during I/R treatment.

### Overexpression of lncRNA136131 can reduce I/R-induced BUMPT cell apoptosis

**Figure 3:**
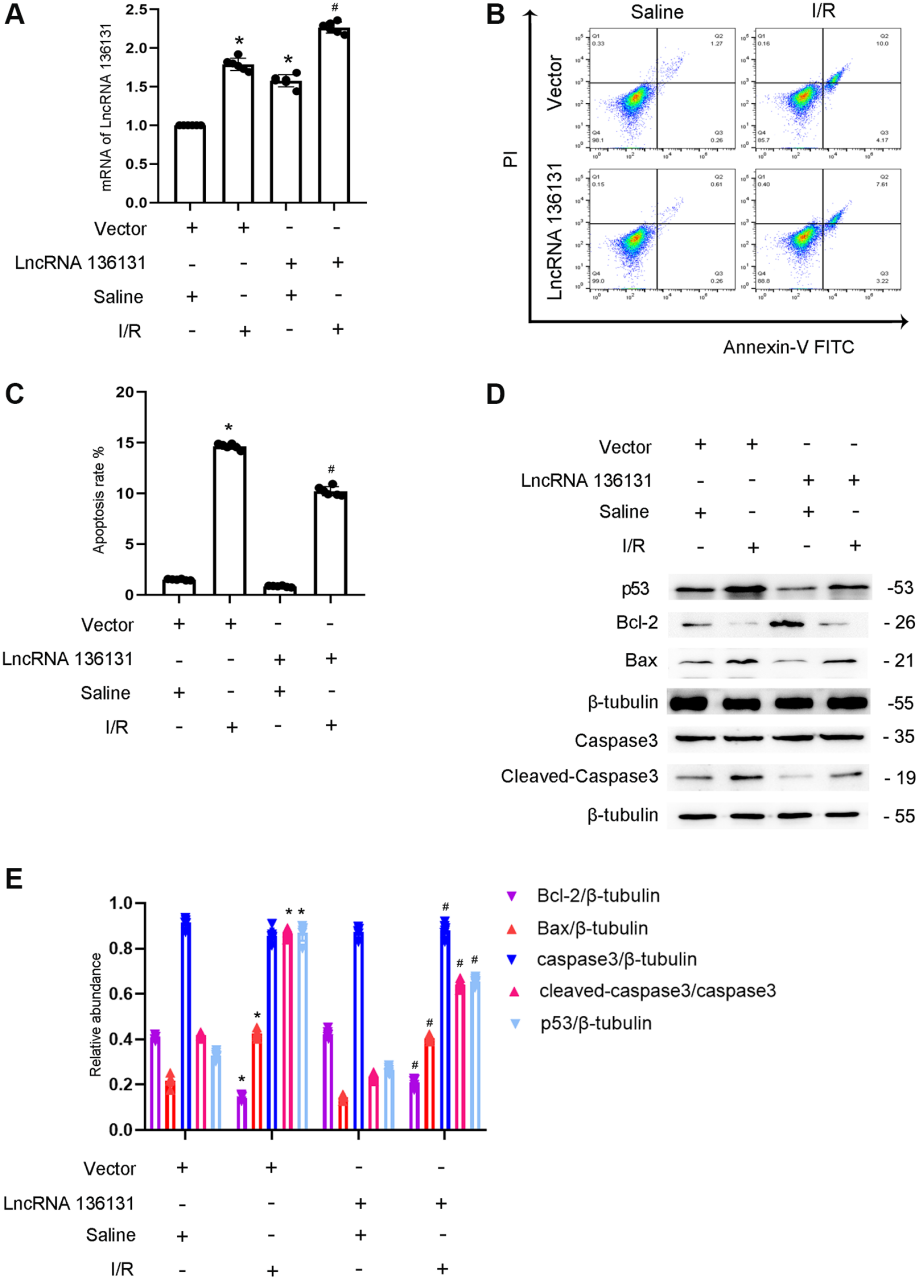
**Overexpression of lncRNA136131 attenuates I/R-induced BUMPT cells apoptosis and the expression of cleaved-caspase3.** BUMPT cells were transfected with lncRNA136131 plasmid or control and then treated with or without I(2 hours)/R(2 hours) injury. (**A**) RT-qPCR analysis of the expression of lncRNA136131. (**B**) FCM analysis of BUMPT cells apoptosis. (**C**) Analysis of apoptosis rate (%). (**D**) The immunoblot analysis of caspase 3, cleaved-caspase3, Bax, p53 and Blc-2. (**E**) The gray analysis between them. Data are expressed as mean ± SD (*n* = 6). ^*^p < 0.05, I/R with scramble or lncRNA136131 siRNA group versus scramble group or Rab10- WT/miR-324-3p mimic versus other groups; ^#^*p* < 0.05, I/R with lncRNA136131 siRNA group versus I/R with scramble group.

As lncRNA136131 has an anti-apoptosis role, we investigated whether the overexpression of lncRNA136131 attenuates apoptosis. The results indicated that overexpression of lncRNA136131 markedly increased its expression under basic or I/R conditions (Figure 3A). FCM analysis demonstrated that overexpression of lncRNA136131 markedly ameliorated I/R-induced BUMPT cells apoptosis (Figure 3B, 3C), which was further supported by immunoblotting cleaved caspase-3,Bax, p53, and Bcl-2 (Figure 3D, 3E). These findings further support the anti-apoptotic effect of lncRNA136131 in BUMPT cells.

### LncRNA136131 inhibits the expression and activity of miR-378a-3p

**Figure 4:**
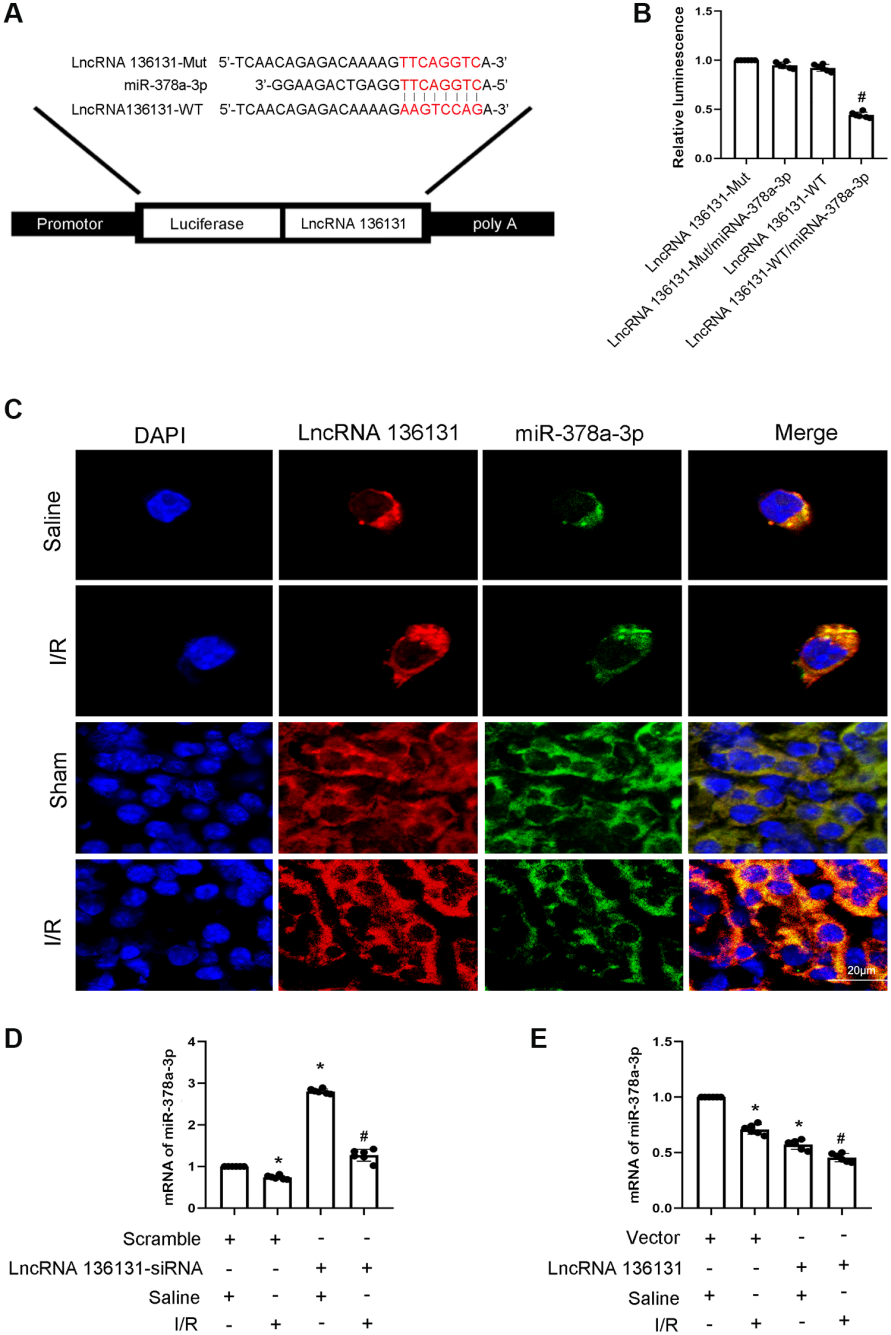
**lncRNA136131 inhibits the expression and activity of miR-378a-3p.** (**A**) Sequence alignment analysis of the complementary strand of lncRNA136131 and miR-378a-3p. (**B**) Detection of luciferase activities after co-transfection with miR-378a-3p plus with lncRNA136131-WT or lncRNA136131-MUT. (**C**) The RNA-FISH detection of Intracellular co-localization of lncRNA136131 and miR-378a-3p in BUMPT cells and mice kidney treated with or without I/R injury. (**D**, **E**) RT-qPCR analysis of miR-378a-3p expression. Data are expressed as mean ± SD (*n* = 6). ^*^p < 0.05, I/R with Scramble group versus Scramble group; ^#^*p* < 0.05, I/R with lncRNA136131 siRNA or plasmid group versus I/R with Scramble group, or lncRNA136131 WT/miR-378a-3p mimic versus other groups.

MiRNAs can be absorbed by LncRNAs. Here, we predicted that miR-378a-3p is a potential downstream target of lncRNA136131 using RegRNA 2.0 software. The prediction results indicated that lncRNA136131 contains the binding site of miR-378a-3p (Figure 4A). Furthermore, luciferase reporter analysis showed that miR-378a-3p mimics inhibited the luciferase activity of lncRNA136131-WT but not lncRNA136131-MUT (Figure 4B). RNA-FISH demonstrated that lncRNA136131 and miR-378a-3p co-localized in the cytoplasm of BUMPT cells and kidney tissue treated with or without I/R injury. (Figure 4C). In addition, I/R-induced inhibition of miR-378a-3p expression was enhanced by lncRNA136131 overexpression, but this effect was reversed by lncRNA136131 knockdown (Figure 4D, 4E). RNA-FISH further confirmed this result (Supplementary Figure 2A, 2B and Supplementary Figure 3A, 3B). In conclusion, the data demonstrate that miR-378a-3p is a direct target of lncRNA136131.

### miR-378a-3p mimics enhance I/R-induced BUMPT cell apoptosis

**Figure 5:**
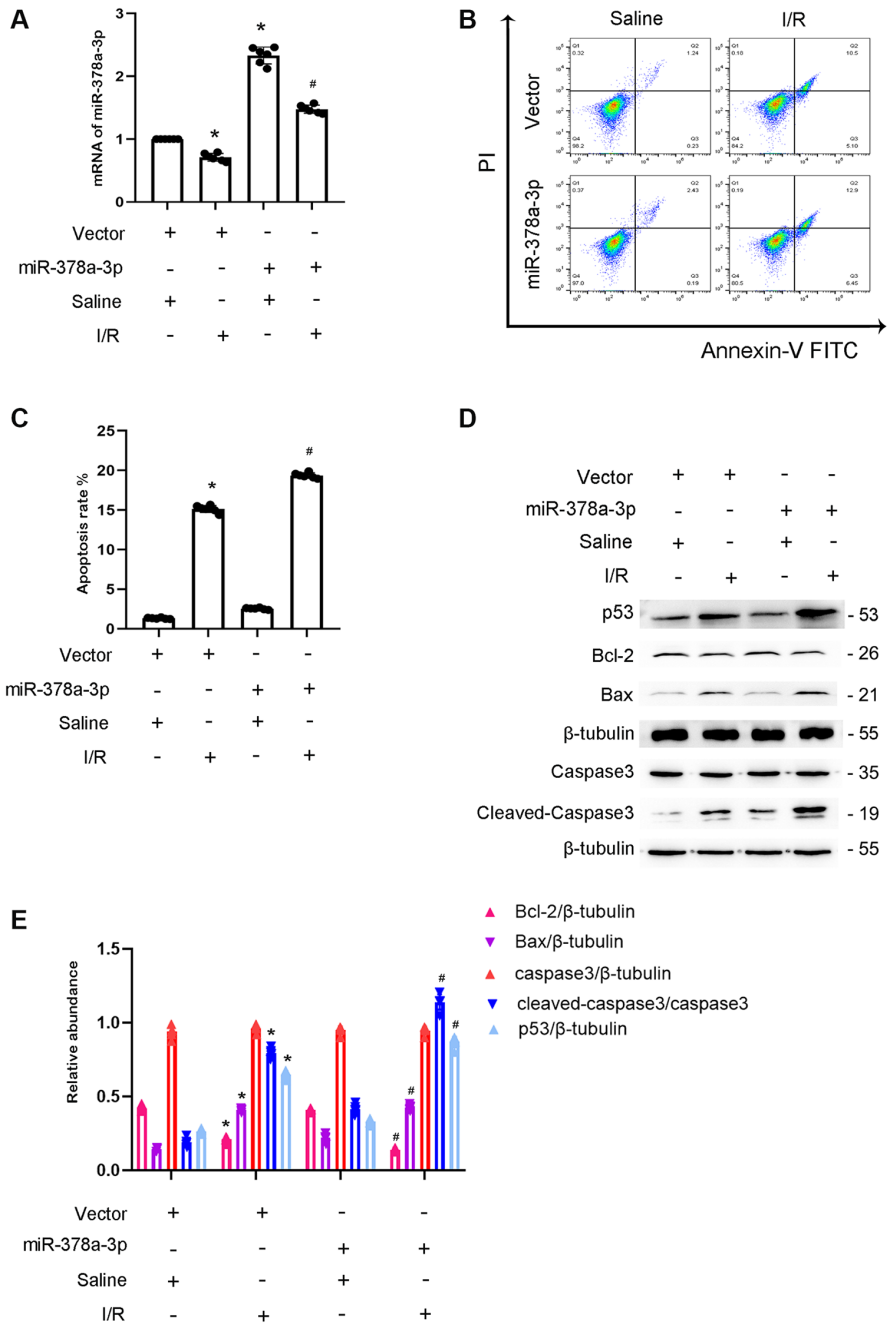
**Overexpression of miR-378a-3p aggravated the I/R-Induced expression levels of cleved-caspase3 in BUMPT cells.** BUMPT cells were transfected with 100 nM miR -378a-3p mimics or scramble and then treated with or without I(2 hours)/R(2 hours) injury. (**A**) The mRNA expression levels of miR-378a-3p were detected by real-time qPCR. (**B**) FCM analysis of BUMPT cells apoptosis. (**C**) Analysis of apoptosis rate (%). (**D**) The immunoblot analysis of caspase 3, cleaved-caspase3, Bax, p53 and Blc-2. (**E**) The gray analysis between them. Data are expressed as mean ± SD (*n* = 6). ^*^p < 0.05, I/R with scramble group versus scramble group; ^#^*p* < 0.05, I/R with miR-378a-3p mimics group versus I/R with scramble group.

Previous studies indicated that miR-378a-3p promotes cardiomyocyte apoptosis in myocardial I/R [16, 17]. We hypothesized that miR-378a-3p mediates I/R-induced BUMPT cells apoptosis. The results indicated that miR-378a-3p mimics increased its mRNA expression (Figure 5A). Flow cytometry analysis showed that miR-378a-3p mimics enhanced I/R-induced BUMPT cells apoptosis (Figure 5B, 5C). In addition, I/R-induced cleaved caspase-3,p53 and Bax expression were also enhanced by the miR-378a-3p mimics, and I/R-induced Bcl-2 expression was reduced by the miR-378a-3p mimics (Figure 5D, 5E). These findings suggest that miR-378a-3p is an apoptosis inducer.

### Rab10 is the direct target gene of miR-378a-3p

**Figure 6:**
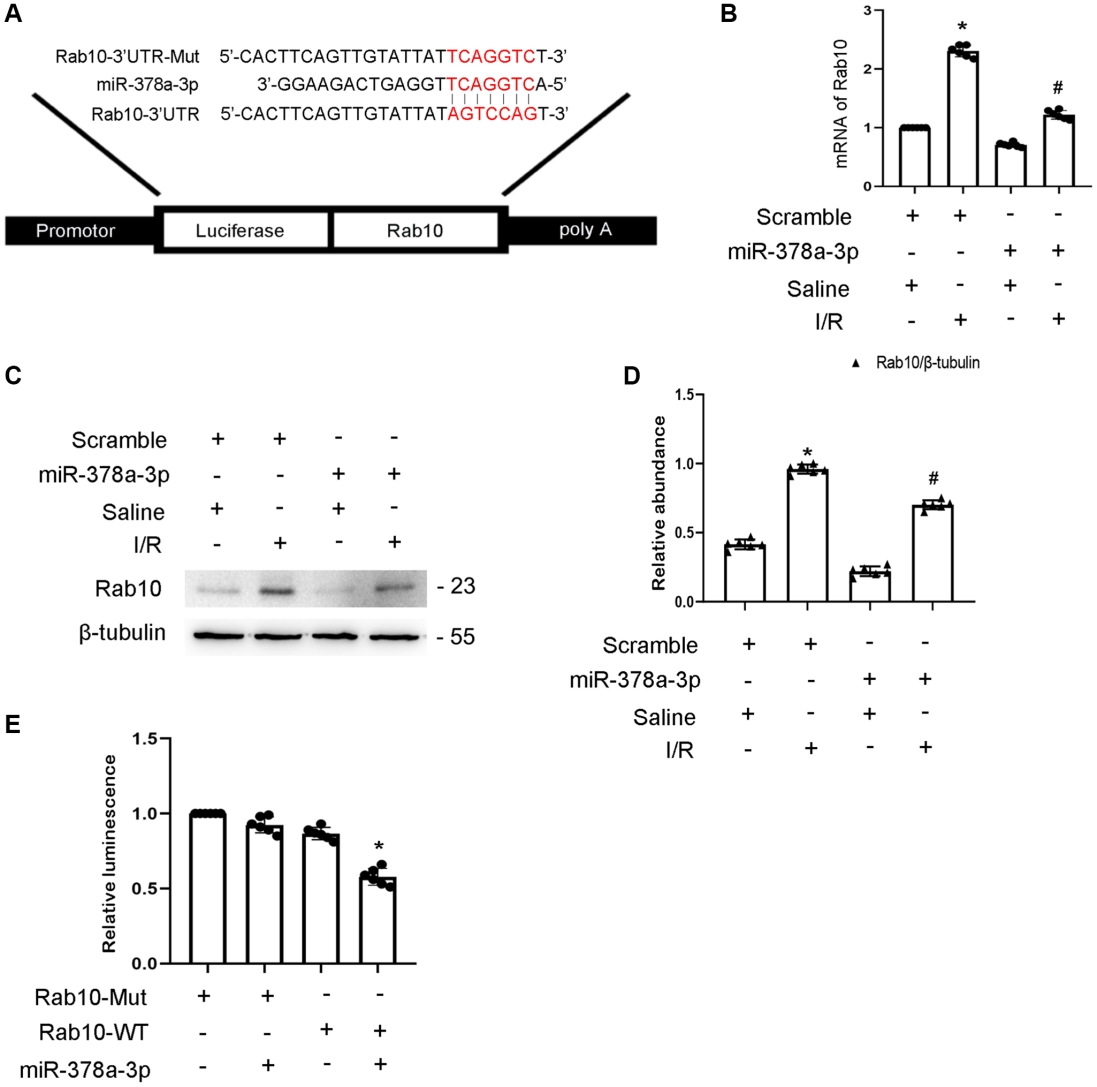
**Rab10 was a direct target of miR-378a-3p.** BUMPT cells were transfected with miR-378a-3p analog (100 nM) and then treated with I(2 hours)/R(2 hours) injury. (**A**) Putative miR-378a-5p complementary binding sites in the 3’UTR of Rab10 mRNA. (**B**) RT-qPCR analysis of the expression of Rab10. (**C**) Immunoblot analysis of Rab10 and β-tubulin. (**D**) Densitometric analysis of proteins signals. (**E**) Detection of luciferase activities after miR-378a-3p co-transfection with the 3’UTR luciferase reporter vector of Rab10-MUT or WT. Data are expressed as mean ± SD (*n* = 6). ^*^p < 0.05, I/R with scramble group versus scramble group; ^#^*p* < 0.05, I/R with miR-378a-3p mimics group versus I/R with scramble group or Rab10 WT/miR-378a-3p mimic versus other groups.

Rab10 is a member of the Rab family of small GTPase. Here, we predict that Rab10 is a target gene of miR-378a-3p using the miRDB website (Figure 6A). In addition, miR-378a-3p mimics significantly suppressed the expression of Rab10 under basic and I/R injury conditions (Figure 6B–6D). The results of the luciferase assay indicated that miR-378a-3p mimics suppressed the luciferase activity of Rab10-WT but not Rab10-MUT (Figure 6E). Taken together, these findings suggest that Rab10 is a target gene of miR-324-3p.

### Silencing of Rab10 enhances I/R-induced apoptosis of BUMPT cells

**Figure 7:**
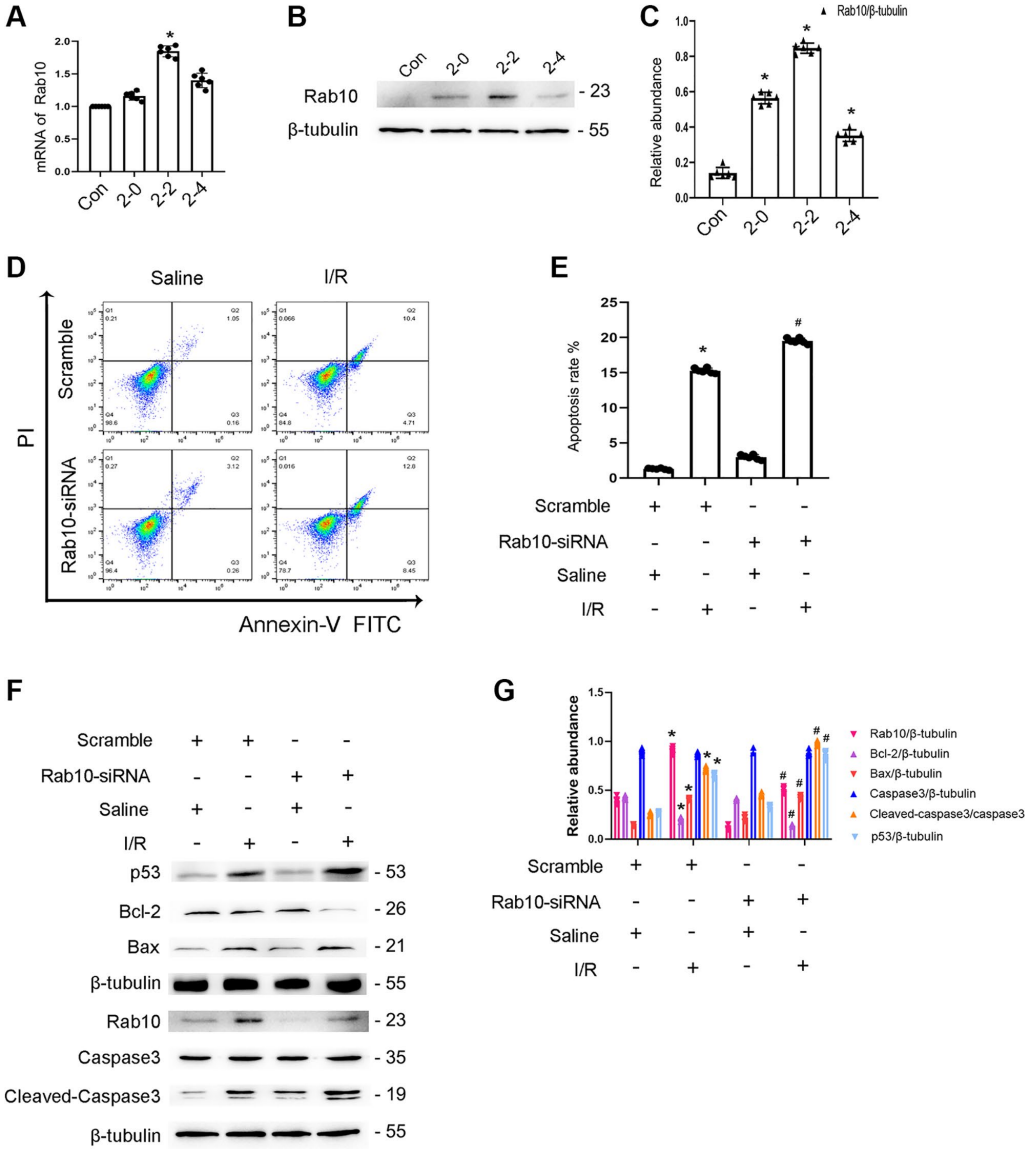
**Knockdown Rab10 aggravates I/R-induced BUMPT cells apoptosis and the expression of cleaved-caspase3.** BUMPT cells were transfected with siRNA Rab10 (100 nM), and then treated with I(2 hours)/R(2 hours) injury. (**A**) RT-qPCR analysis of the expression of Rab10. (**B**) Immunoblot analysis of Rab10 and β-tubulin at indicated time points. (**C**) Densitometric analysis of proteins signals. (**D**) FCM analysis of BUMPT cells apoptosis. (**E**) The apoptosis rate (%). (**F**) Immunoblot analysis of caspase 3, cleaved-caspase3, Bax, Blc-2, p53 and Rab10. (**G**) Densitometric analysis of proteins signals. Data are expressed as mean ± SD (*n* = 6). ^*^p < 0.05, I/R with scramble group or I/R group versus scramble group or control group; ^#^*p* < 0.05, I/R with siRNA Rab10 group versus I/R with scramble group.

The role of Rab10 in renal tubular cell apoptosis remains unclear. Initially, RT-qPCR analysis indicated that the expression of Rab10 induced by ischemic injury gradually increased at 0 h after reperfusion, peaked 2 h after reperfusion, and finally decreased 4 h after reperfusion (Figure 7A), which coincided with the expression profile of Rab10 (Figure 7B, 7C). Additionally, Rab10 siRNA enhanced I/R-induced BUMPT cell apoptosis (Figure 7D, 7E), which was further verified by immunoblotting of cleaved caspase-3,Bax, p53, and Bcl-2 (Figure 7F, 7G). These findings suggest that Rab10 is an anti-apoptosis protein.

### I/R-induced BUMPT cell apoptosis is ameliorated by Rab10 overexpression

**Figure 8:**
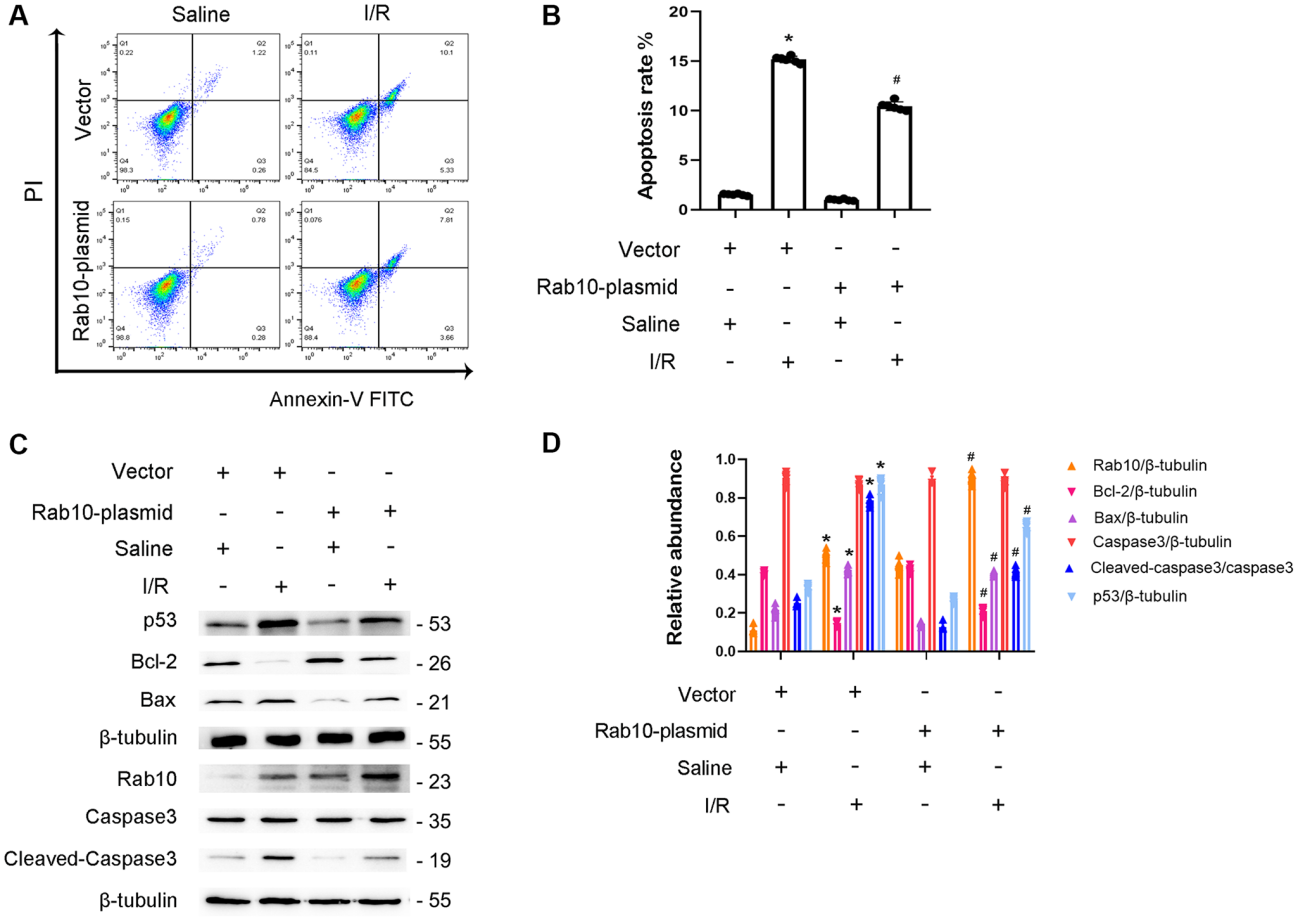
**Overexpression of Rab10 attenuated I/R-induced BUMPT cells apoptosis and the expression of cleaved-caspase3.** Rab10 plasmid (100 nM) was transfected into BUMPT cells, and then treated with I(2 hours)/R(2 hours). (**A**) FCM analysis of BUMPT cells apoptosis. (**B**) The apoptosis rate (%). (**C**) Immunoblot analysis of caspase 3, cleaved-caspase3, Bax, Blc-2, p53 and Rab10. (**D**) Densitometric analysis of proteins signals. Data are expressed as mean ± SD (*n* = 6). ^*^p < 0.05, I/R with scramble group or I/R group versus scramble or control group ; ^#^*p* < 0.05, I/R with Rab10 plasmid group versus I/R with scramble group.

To confirm the role of Rab10, Rab10 plasmids were transfected into BUMPT cells. After I/R injury, FCM analysis showed that the overexpression of Rab10 significantly reduced I/R-induced BUMPT cell apoptosis (Figure 8A, 8B). In addition, overexpression of Rab10 suppressed the I/R-induced expression of cleaved caspase-3,p53 and Bax and increased the expression of Bcl-2 (Figure 8C, 8D). In conclusion, our findings further support that Rab10 plays an anti-apoptotic role during I/R injury.

### miR-378a-3p inhibitor reverses the effect of lncRNA136131 knockdown on I/R-Induced BUMPT cell apoptosis

**Figure 9:**
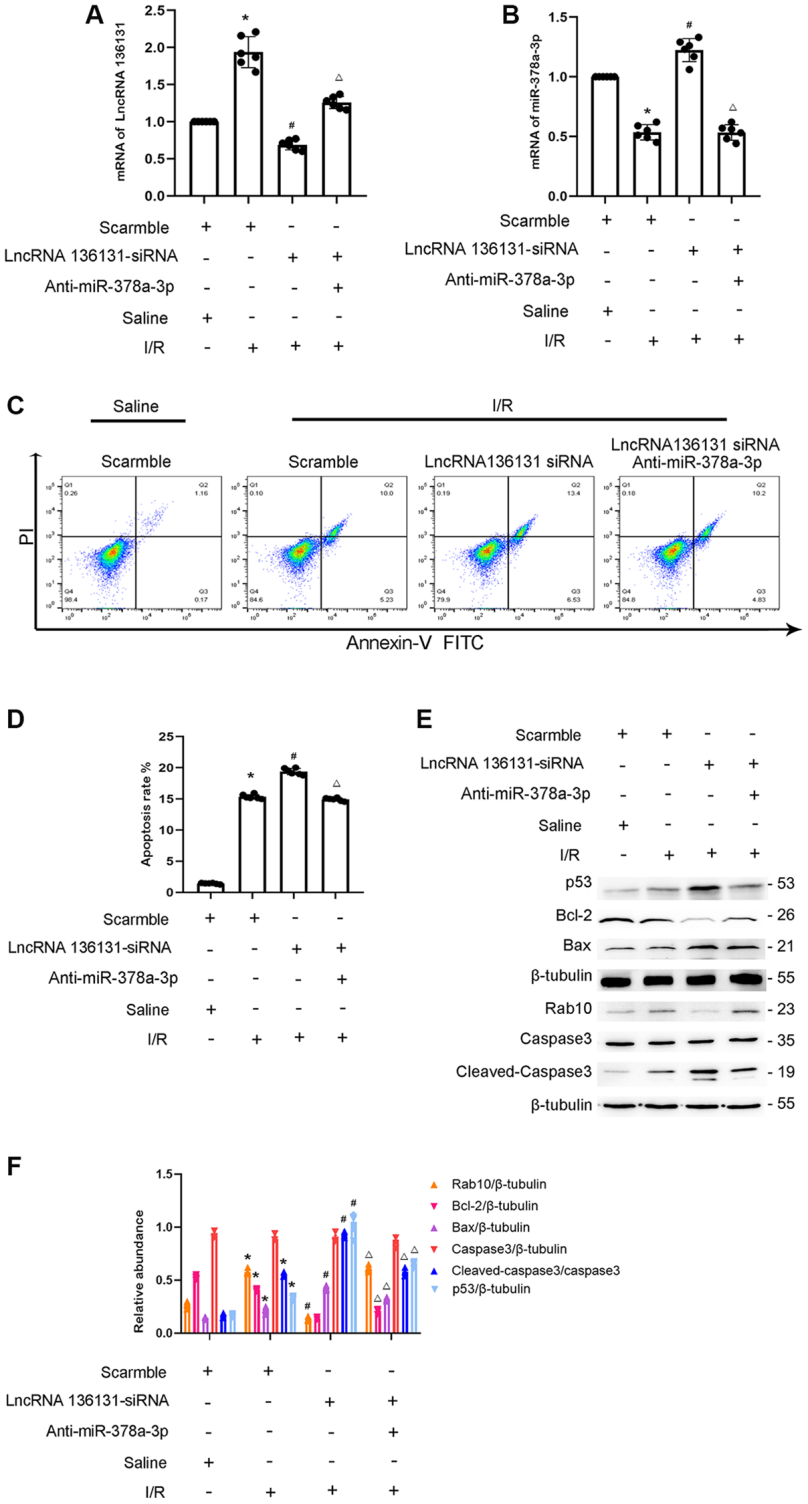
**LncRNA136131 knockdown aggravated the I/R-Induced BUMPT cells apoptosis and the expression of cleaved caspase3, this was reversed by miR-378a-3p inhibitor.** BUMPT cells were co-transfected with siRNA lncRNA136131 (100 nM) plus with or without anti-miR-378a-3p, and then treated with I(2 hours)/R(2 hours) injury. (**A**) RT-qPCR analysis the expression of lncRNA136131. (**B**) Real-time qPCR analysis of miR-378a-3p expression. (**C**) FCM analysis of BUMPT cells apoptosis. (**D**) Cell apoptosis rate (%). (**E**) Immunoblot analysis of caspase 3, cleaved-caspase3, Bax, Blc-2, p53 and Rab10. (**F**) Densitometric measurement of protein signals. Data are expressed as mean ± SD (*n* = 6). ^*^p < 0.05, I/R with scramble group versus scramble group, ^#^*p* < 0.05, I/R with lncRNA136131 siRNA group versus I/R with scramble group; ^Δ^*p* < 0.05, I/R with lncRNA136131 plus anti-miR-378a-3p group versus I/R with lncRNA136131 siRNA group.

We investigated whether miR-378a-3p mediates the anti-apoptosis effect of lncRNA136131 in the I/R process. RT-qPCR analysis indicated that siRNA lncRNA136131 and miR-378a-3p were effective in BUMPT cells (Figure 9A, 9B). FCM analysis found that lncRNA136131 knockdown aggravated I/R-induced BUMPT cell apoptosis, and this effect was reversed by the miR-378a-3p inhibitor (Figure 9C, 9D). Immunoblot analysis showed that lncRNA136131 siRNA increased the expression level of I/R-induced cleaved caspase-3,p53 and Bax and induced the expression of Bcl-2. This effect was reversed by miR-378a-3p inhibitor (Figure 9E and 9F). These findings provide strong evidence that miR-378a-3p mediates anti-apoptosis of lncRNA136131 during I/R injury.

### Overexpression of lnc136131 alleviates I/R-Induced AKI by targeting the miR-378a-3p/Rab10 axis

To further confirm the role of lnc136131 in ischemic AKI, lnc136131 expressing vectors were injected into C57BL/6J mice via tail vein 12 h before I/R treatment. First, the overexpression of lnc136131 notably prevented the I/R-induced decline in renal function, tubular damage, renal cell apoptosis, and upregulated expression of Rab10 (Supplementary Figure 4A–4H). Furthermore, the expression levels of Lnc136131 and miR-378a-3p were reversed by overexpression of lncRNA136131 under normal and ischemic treatment conditions (Supplementary Figure 4I and 4J). Immunoblot analysis demonstrated that I/R induced the upregulated expression of caspase-3, cleaved caspase-3,p53, and Bax, whereas reduced the expression of Bcl-2 and Rab10 was reduced by the overexpression of lncRNA136131 (Supplementary Figure 4K and 4L). These findings indicate that the lncRNA136131/miR-378a-3p/Rab10 axis may be involved in AKI.

### I/R-induced AKI is aggravated by knocking down lncRNA136131 by targeting the miR-378a-3p/Rab10 axis

To verify the role of lncRNA136131 in ischemic AKI, lncRNA136131 siRNA was injected into C57BL/6J mice via tail vein 12 h before I/R treatment. First, I/R reduced renal function, tubular damage, and renal cell apoptosis, and the expression of Rab10 was enhanced by the lncRNA136131 siRNA (Supplementary Figure 5A–5H). LncRNA136131 siRNA reversed the expression of lnc136131 and miR-378a-3p under normal and ischemic treatment conditions (Supplementary Figure 5I and 5J). Immunoblot analysis showed that I/R increased the expression of caspase-3, cleaved caspase-3,p53, and Bax, whereas the downregulation of Bcl-2 and Rab10 was enhanced by lncRNA136131 siRNA (Supplementary Figure 5K and 5L). In conclusion, these findings further support that the lncRNA136131/miR-378a-3p/Rab10 axis imparted a protective effect against ischemic AKI.

## DISCUSSION

This study, for the first time, demonstrated that lncRNA136131 is induced by I/R treatment. Furthermore, lncRNA136131 imparted a protective effect against I/R-induced apoptosis of renal tubular epithelial cells. Mechanistically, lncRNA136131 binds to miR-378a-3p and then increases Rab10 expression to prevent apoptosis. Finally, overexpression of lncRNA136131 attenuated the progression of I/R-induced ischemic AKI.

Previous studies have demonstrated that lncRNAs play an important role in renal fibrosis [14, 18–21]. However, more recent studies have shown that lncRNAs are involved in renal cell apoptosis. For example, lncRNA TCONS-00016406, lncRNA LOC105374325, and lncRNA TCONS 00016233 mediate LPS-induced renal cell apoptosis [9, 10, 13]. However, lncRNA PRNCR1 prevented cisplatin-induced renal epithelial cells apoptosis [11]. In the current study, we found that lncRNA136131 plays an anti-apoptosis role during ischemic injury, which was supported by the following evidence. *In vitro*, I/R-induced BUMPT cell apoptosis was enhanced by lncRNA136131 siRNA, but was attenuated by the overexpression of lncRNA136131 (Figures 2 and 3). *In vivo*, I/R-induced renal tubular cell apoptosis was enhanced by lncRNA136131 siRNA, but was ameliorated by the overexpression of lncRNA136131 (Supplementary Figures 4 and 5). Collectively, these findings suggest that lncRNA136131 plays an anti-apoptotic role during ischemic injury.

More evidence suggests that lncRNAs act as ceRNAs that regulate target gene expression [13, 14, 21, 22]. In the current study, we found that miR-378a-3p is a target of lncRNA136131. The following findings provide strong support for this finding. First, dual luciferase reporter analysis demonstrated that lncRNA136131 directly binds to miR-378a-3p (Figure 4). Second, FISH colocalization analysis indicated that lncRNA136131 interacts with miR-378a-3p in the cytoplasm of BUMPT cells and renal tubular cells of mouse kidneys under basic and I/R treatment conditions (Figure 4). Finally, qRT-PCR analysis revealed that the expression of miR-378a-3p is reduced by the overexpression of lncRNA136131 and was increased by lncRNA136131 siRNA under basic and I/R treatment conditions (Figure 4). In conclusion, these findings support that miR-378a-3p is a direct target of lncRNA136131.

To date, the role of miR-378a-3p in cell apoptosis remains unclear. Two studies have found that miR-378a-3p promotes the apoptosis of ovarian cancer cells and retinoblastoma cells [17, 23]. However, one study demonstrated that miR-378a-3p prevents H9C2 cardiomyocyte cells apoptosis during ischemic injury [24]. Furthermore, another study reported that miR-378a-3p induces Ferroptosis in rat ischemic kidney injury [25]. However, current understanding of the role of miR-378a-3p in renal cell apoptosis remains limited. In this study, the overexpression of miR-378a-3p exacerbated I/R-induced renal cell apoptosis (Figure 5), which suggests that miR-378a-3p promotes renal cell apoptosis during ischemic injury.

In addition, Rab10, a member of the Rab family of small GTPases [26], was identified as a target of miR-378a-3p and is supported by the following evidence. Dual luciferase reporter analysis supports that miR-378a-3p interacts with Rab10 (Figure 6). Overexpression of miR-378a-3p significantly suppressed Rab10 expression under basic and I/R treatment (Figure 6). This is consistent with the finding that Rab10 is a downstream gene of miR-378a-3p and is associated with tumor growth [27]. Although some studies have reported that Rab10 plays an anti-apoptotic role in tumors [28–30], the effect of Rab10 in renal cell apoptosis remains unclear. First, Rab10 was induced by I/R treatment (Figure 7). Second, I/R-induced renal tubular cell apoptosis was attenuated by Rab10 siRNA; in contrast, this was enhanced by the overexpression of Rab10. In addition, we also verified that lncRNA136131 knockdown increased I/R-induced apoptosis by targeting Rab10; this effect was reversed by an miR-378a-3p inhibitor (Figure 7). These findings suggest that Rab10 is a direct target of lncRNA136131.

In conclusion, our results show that I/R induces lncRNA136131, which protects against renal cell apoptosis during ischemic injury. Mechanistically, lncRNA136131 binds to miR-378a-3p and then increases the expression of Rab10 to suppress renal cell apoptosis. Furthermore, overexpression of lncRNA136131 attenuates I/R-induced progression of AKI. In conclusion, the lncRNA136131/miR-378a-3p/Rab10 axis plays a renal protective role during ischemic injury, which suggests that overexpression of lncRNA136131may become a novel therapeutic regimen for AKI.

## MATERIALS AND METHODS

### Antibodies and reagents

Antibody Rab10 (Cat. No. 11808-1-AP) and Antibody p53 (Cat. No.60283-2-Ig) were obtained from ProteinTech North America (Rosemont, IL, USA). Anti-cleaved caspase-3 (Cat. No. 9661s) was purchased from Cell Signaling Technology (Danvers, MA, USA). Anti-caspase-3 (Cat. No. ab184787) was obtained from Abcam (Cambridge, MA, USA). Anti-β-tubulin (Cat. No. T0023) was purchased from Affinity Biosciences (Cincinnati, OH, USA). Anti-bax (Cat. No.AF120) and bcl2 (Cat. No. AF6139) was purchased from Affinity Biosciences (Cincinnati, OH, USA). The luciferase assay kit was obtained from BioVision (Milpitas, CA, USA). Anti-β-Tubulin (Cat. No. 86298) was purchased from Cell Signaling Technology (Danvers, MA, USA).

### Cell culture and treatments

BUMPT cells were cultured in DMEM (Sigma-Aldrich) supplemented by 10% fetal bovine serum and antibiotics (100 U/mL penicillin G and 100 μg/mL streptomycin) at 37°C in a humidified atmosphere of 5% CO_2_ and 95% air. When BUMPT cells were 90% confluent, the culture medium was replaced with Hank’s balanced salt solution (HBSS; containing 1.3 mM Ca^++^ and 0.8 mM Mg^++^) plus with antimycin A (Shanghai Maokang Biotechnology Co., Ltd. No: 1397-94-0) and calcium ionophore (Shanghai Aladdin Biotechnology Co., Ltd. CAS NO 52665-69-7) treatment for 0–2 h, and then allowed to recover for 0–4 h. For gene interference experiments, the cells were incubated siRNA with lncRNA136131 (100 nM), lncRNA136131 plasmid, miR-378a-3p mimic (100 nM), miR-378a-3p inhibitor (100 nM), Rab10 siRNA (100 nM), Rab10 plasmid, or the negative control (Ruibo, Guangzhou, China) using Lipofectamine 2000 (Life Technologies, Carlsbad, CA, USA).

### Ischemic AKI model

C57BL/6J mice were purchased from Shanghai Animal Center (Shanghai, People’s Republic of China). Animal experiments were conducted following the guidelines of the Animal Care Ethics Committee of Second Xiangya Hospital, People’s Republic of China. The mice were housed in a 12-h light/dark cycle and had free access to food and water. C57BL/6J mice (males, aged 10–12 weeks) were pre-injected with lncRNA136131 siRNA (15 mg/kg per injection) [31] or lncRNA136131 plasmid (25 μg of DNA per injection) [13] -expressing vectors through the tail vein and saline as control. The bilateral renal artery was continuously clipped for 28 min and then released for 24 h or 48 h as previously described [32].

### RT-qPCR analysis

According to the manufacturer’s instruction, TRIzol reagent (Invitrogen, Carlsbad, CA, USA) was used to extract total RNA from BUMPT cells and mouse kidney cortex. About 2 μg of the total RNA was reverse transcribed using Evo M-MLV reverse transcriptase. RT-qPCR was performed by Bio-Rad (Hercules, CA, USA) Ag SYBR Green Pro Taqhs premix (Accurate Biotechnology, China) following the manufacturer’s protocol. The sequences of lncRNA136131 were retrieved from the Ensembl database (Gen ID: ENSMUST00000136131.2). The primers sequences were as follows: LncRNA136131, 5′-AGCAGTCTGAAGGCACAGCA-3′ (forward) and 5′-CTGGT ACACCCACCACTGGT-3′ (reverse); miR-378a-3p, 5′-CGCGACTGGACTTGGAGTC-3′ (forward) and 5′-AGTGCAGGGTCCGAGGTATT-3′ (reverse); Rab10, 5′-GCTGAAGACATCCTCCGAAAGACC-3′ (forward) and 5′-CCGTCACGCCTCCTCCACT G-3′ (reverse); β-actin, 5′-GGCTGTATTCCCCTCCATCG-3′ (forward) and 5′-CCAGTTGGTAACAATGCCATGT-3′ (reverse); U6, 5′-CTCGCTTCGGCAGCACA-3′ (forward) and 5′-AACGCTTCACGAATTTGCGT-3′ (reverse). The relative quantification was performed by determining ΔCt values.

### FISH analysis

The fluorescent probes of lncRNA136131 and miR-378a-3p were synthesized by RiBo company (Guangzhou, China). The 40,6-diamidino-2-phenylindole (DAPI) was used to stain the nuclei of BUMPT cells, U6 for nuclear staining, and 18S rRNA for cytoplasmic staining. LncRNA136131 was labeled with Cy3. BUMPT cells and mouse kidney slides were hybridized with the corresponding probes overnight, and then stained with DAPI. The fluorescence imaging was performed with a laser scanning confocal microscope.

### Luciferase reporter assays

Luciferase reporter assays were conducted as previously described [33–35]. The luciferase gene (luc2) pmirGLO double luciferase expression vector containing the miRNA target gene, responsive elements of Rab10-3′UTR (WT Luc Rab10), Rab10 (MUT Luc Rab10), or lncRNA136131 (WT Luc lncRNA136131) or lncRNA136131 (MUT Luc lncRNA136131) with the responsive elements of miR-378a-3p were used. The control vector was used with the PGMLR-TK luciferase reporter. All plasmids were established by Ricky Biology (Guangzhou, Guangdong, China). Briefly, the plasmids of WT Luc, MUT Luc, or PGMLR-TK were co-transfected with miR-378a-3p mimics into BUMPT cells for 48 h. Then, luciferase activity was examined by SpectraMax M5 (Molecular Devices, Sunnyvale, CA, USA) and normalized to pGMLR-TK activity.

### Renal function, morphology, and apoptosis

Renal function was evaluated using blood urea nitrogen and creatinine levels in accordance with the instructions provided in the kits (Nanjing Jiancheng Bioengineering Institute, Jiangsu, China). H&E staining was used to assess tissue damage [36, 37]. TUNEL staining was performed to evaluate renal cell apoptosis. The percentage of positive cells was counted as described in a previous study [38]. Rab10 (1:50 dilution) was used for immunohistochemical analysis [39]. A fluorescein isothiocyanate (FITC) Annexin V Apoptosis Detection Kit I (Cat. No. 556547; BD Pharmingen, Franklin, NJ, USA) was employed.

### Immunoblot analysis

The same amount of protein was loaded on each channel, and then separated by SDS-PAGE. The separated protein was transferred onto a nitrocellulose membrane (Amersham, Buckinghamshire, UK) [38, 40] after electrophoresis. Then, the membrane was examined with the primary antibodies against Rab10, cleaved caspase-3, and caspase-3, and then incubated with the respective secondary antibodies and detection reagents. β- tubulin was used as internal control.

### Statistical analysis

Two-tailed student *t* tests were used to assess differences between the two groups. Multiple group comparisons were conducted by one-way ANOVA. Quantitative data were expressed as the mean ± SD. Differences with *p* < 0.05 were considered statistically significant.

## Supplementary Materials

Supplementary Figures
