# Supplementary material for: LncRNA136131 suppresses apoptosis of renal tubular epithelial cells in acute kidney injury by targeting the miR-378a-3p/Rab10 axis
Source: Aging (Albany NY). 2022 Apr 28;14(8):3666–86. doi: 10.18632/aging.204036 (PMC9085219; doi:10.18632/aging.204036)
Supplement: Supplementary Figures [file aging-14-204036-s001.pdf]

SUPPLEMENTARY FIGURES

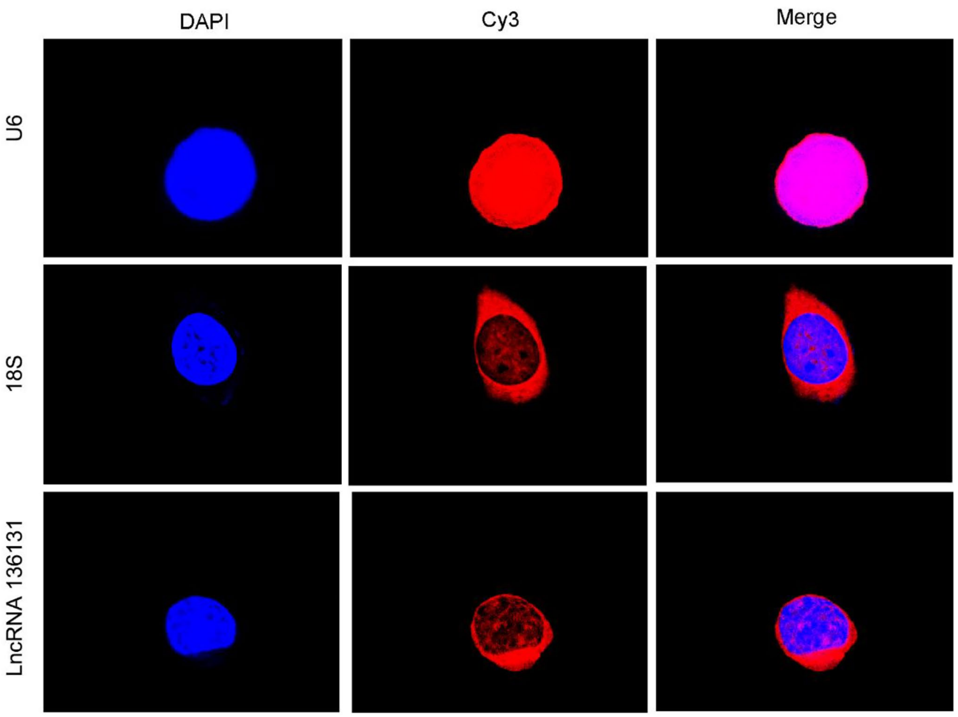

Supplementary Figure 1. The RNA-FISH to verify the lncRNA136131 located in the cytoplasm of BUMPT cells. Repeat the experiment of LncRNA13631 located in the cytoplasm of BUMPT cells.

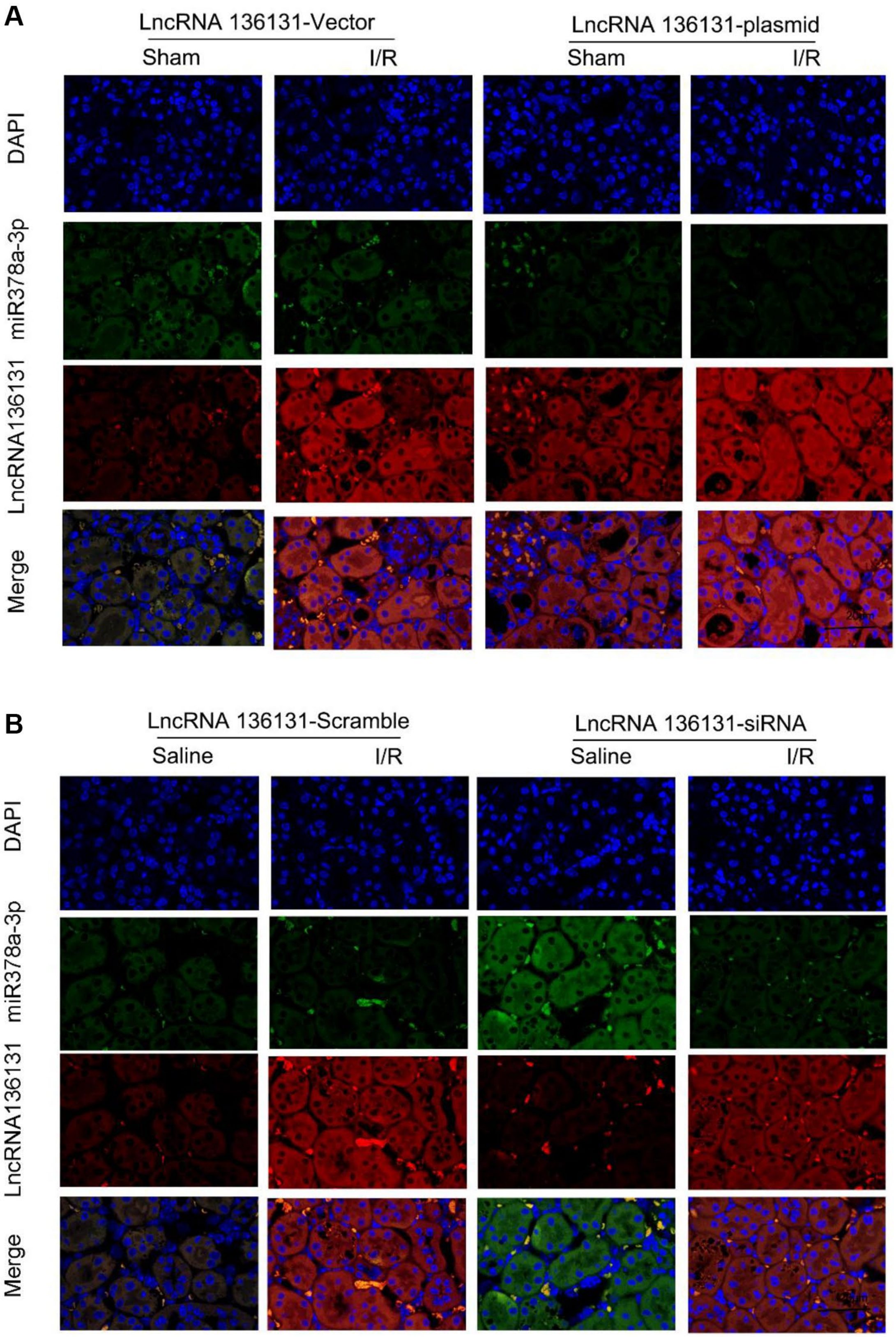

Supplementary Figure 2. The RNA-FISH to verify the effect of lncRNA136131 overexpression and knockdown on miR-378a-3p in kidney tissue. (**A**) After knockdown of lncRNA136131, The RNA-FISH detected the expression of lncRNA136131 and miR-378a-3p in mice kidney treated with or without I/R injury. (**B**) After overexpression of lncRNA136131, The RNA-FISH detected the expression of lncRNA136131 and miR-378a-3p in mice kidney treated with or without I/R injury.

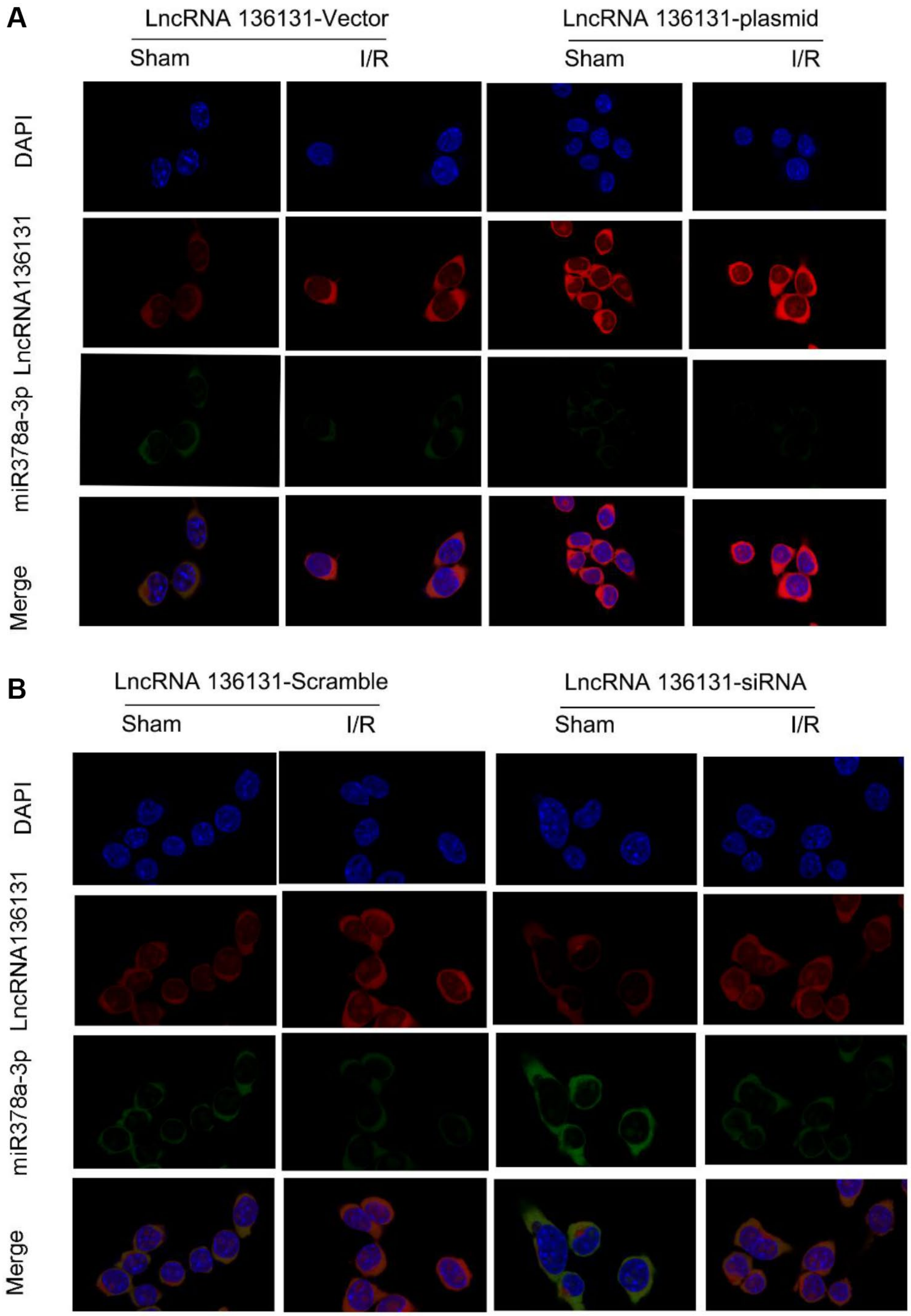

Supplementary Figure 3. The RNA-FISH to verify the effect of lncRNA136131 overexpression and knockdown on miR-378a-3p in BUMPT cells. (**A**) After knockdown of lncRNA136131, The RNA-FISH detected the expression of lncRNA136131 and miR-378a-3p in BUMPT cells treated with or without I/R injury. (**B**) After overexpression of lncRNA136131, The RNA-FISH detected the expression of lncRNA136131 and miR-378a-3p in BUMPT cells treated with or without I/R injury.

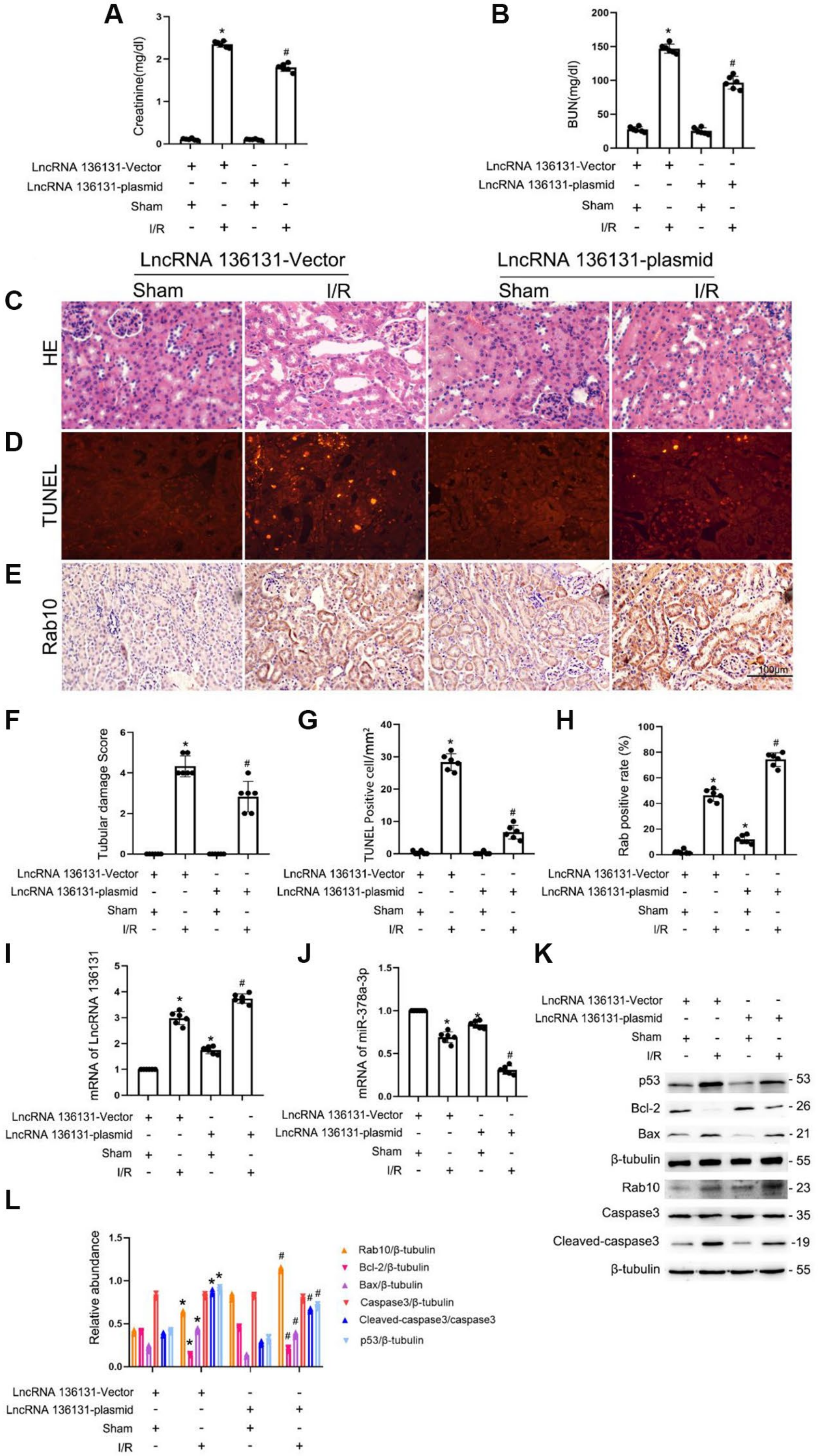

Supplementary Figure 4. Overexpression of lnc136131 ameliorated I/R-Induced AKI. The C57BL/6J mice were injected with lnc136131 plasmid via tail vein for 12 h, and then the bilateral renal pedicles were clipped for 28 minutes and subsequently reperfused for 48 hours. Blood serum was obtained for detection of creatinine (**A**) and nitrogen (BUN) (**B**) concentration. Renal tissues were stained with hematoxylin and eosin (H&E) (**C**), TUNEL (**D**), and immunohistochemical staining of Rab10 (**E**). (**F**) Tubular damage scores of kidney cortex. (**G**) Counting of TUNEL-positive cells (**H**) Quantification of immunohistochemical staining. (**I**, **J**) RT-qPCR analysis of the expression of lncRNA136131 and miR-378a-3p. (**K**) Immunoblot analysis of caspase 3, cleaved-caspase3, Bax, Blc-2, p53 and Rab10. (**L**) Densitometric analysis of immunoblot bands. Original magnification, ×400. Data are expressed as mean ± SD (n = 6). $^*p < 0.05$, I/R with control group versus sham group; $^{\#}p < 0.05$, I/R group with Lnc136131 plasmid versus I/R with control group.

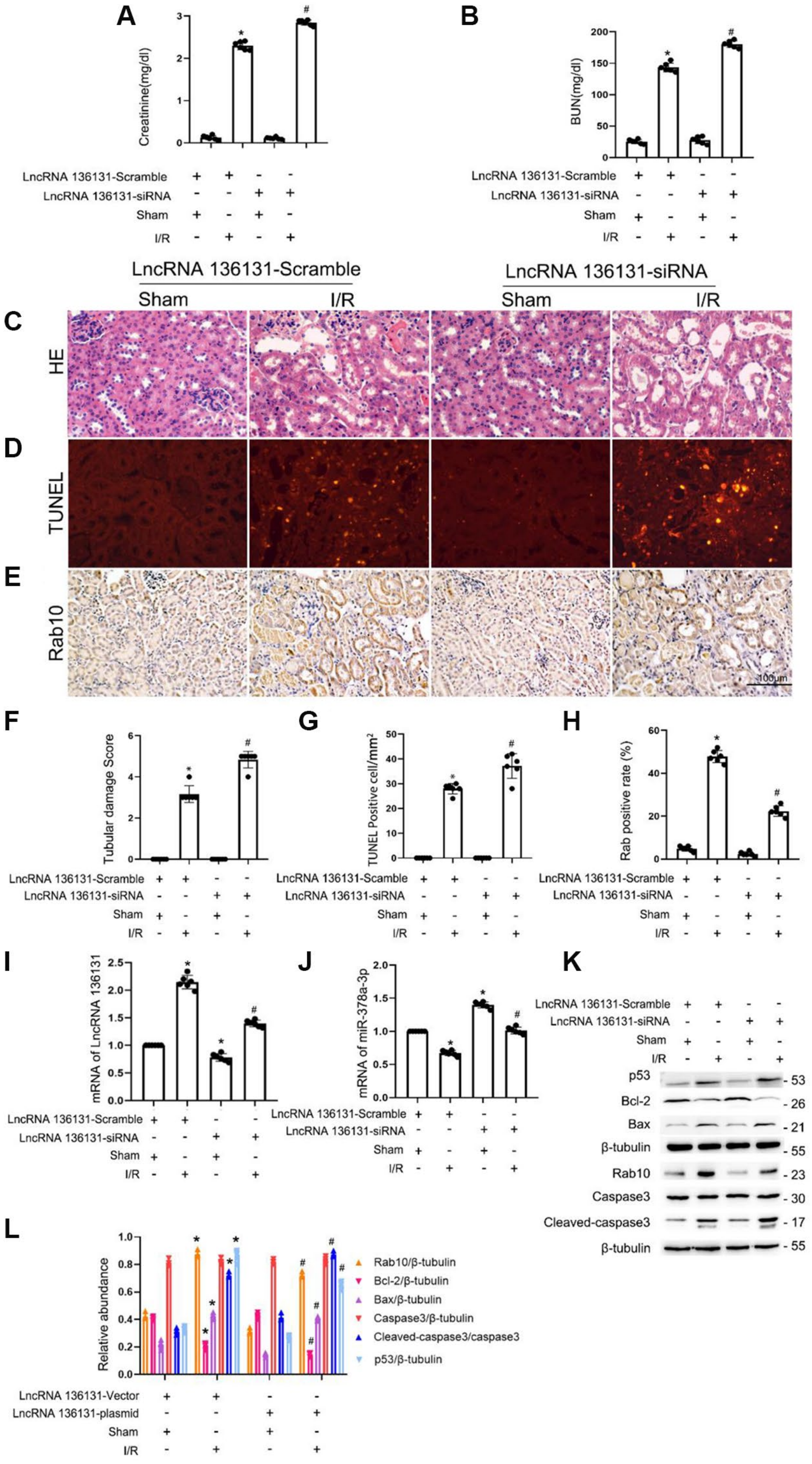

Supplementary Figure 5. Knockdown of lncRNA136131 aggravated I/R-Induced AKI. The C57BL/6J mice were injected with lncRNA136131 siRNA via tail vein for 12 h, and then the bilateral renal pedicles were clipped for 28 minutes and subsequently reperfused for 48 hours. Blood serum was obtained for detection of creatinine (**A**) and nitrogen (BUN) (**B**) concentration. Renal tissues were stained with hematoxylin and eosin (H&E) (**C**), TUNEL (**D**), and immunohistochemical staining of Rab10 (**E**). (**F**) Tubular damage scores of kidney cortex. (**G**) Counting of TUNEL-positive cells. (**H**) Quantification of immunohistochemical staining. (**I**, **J**) RT-qPCR analysis of the expression of lncRNA136131 and miR-378a-3p. (**K**) Immunoblot analysis of caspase 3, cleaved-caspase3, Bax, Blc-2, p53 and Rab10. (**L**) Densitometric analysis of immunoblot bands. Original magnification, ×400. Data are expressed as mean ± SD (*n* = 6). $^{*}p < 0.05$, I/R with control group versus sham group; $^{\#}p < 0.05$, I/R group with Lnc136131 siRNA versus I/R with control group.
